# Lack of Health Insurance Among Adults Aged 18 to 64 Years: Findings From the 2013 Behavioral Risk Factor Surveillance System

**DOI:** 10.5888/pcd12.150328

**Published:** 2015-12-31

**Authors:** Catherine A. Okoro, Guixiang Zhao, Satvinder S. Dhingra, Fang Xu

**Affiliations:** Author Affiliations: Guixiang Zhao, Fang Xu, Population Health Surveillance Branch, Division of Population Health, National Center for Chronic Disease Prevention and Health Promotion, Centers for Disease Control and Prevention, Atlanta, Georgia; Satvinder S. Dhingra, Northrop Grumman Corporation, Atlanta, Georgia.

## Abstract

**Introduction:**

The objective of this study was to estimate the prevalence of lack of health insurance among adults aged 18 to 64 years for each state and the United States and to describe populations without insurance.

**Methods:**

We used 2013 Behavioral Risk Factor Surveillance System data to categorize states into 3 groups on the basis of the prevalence of lack of health insurance in each state compared with the national average (21.5%; 95% confidence interval, 21.1%–21.8%): high-insured states (states with an estimated prevalence of lack of health insurance below the national average), average-insured states (states with an estimated prevalence of lack of health insurance equivalent to the national average), and low-insured states (states with an estimated prevalence of lack of health insurance higher than the national average).

We used bivariate analyses to compare the sociodemographic characteristics of these 3 groups after age adjustment to the 2000 US standard population. We examined the distribution of Medicaid expansion among the 3 groups.

**Results:**

Compared with the national age-adjusted prevalence of lack of health insurance, 24 states had lower rates of uninsured residents, 12 states had equivalent rates of uninsured, and 15 states had higher rates of uninsured. Compared with adults in the high-insured and average-insured state groups, adults in the low-insured state group were more likely to be non-Hispanic black or Hispanic, to have less than a high school education, to be previously married (divorced, widowed, or separated), and to have an annual household income at or below $35,000. Seventy-one percent of high-insured states were expanding Medicaid eligibility compared with 67% of average-insured states and 40% of low-insured states.

**Conclusion:**

Large variations exist among states in the estimated prevalence of health insurance. Many uninsured Americans reside in states that have opted out of Medicaid expansion.

## MEDSCAPE CME

Medscape, LLC is pleased to provide online continuing medical education (CME) for this journal article, allowing clinicians the opportunity to earn CME credit.

This activity has been planned and implemented in accordance with the Essential Areas and policies of the Accreditation Council for Continuing Medical Education through the joint sponsorship of Medscape, LLC and *Preventing Chronic Disease*. Medscape, LLC is accredited by the ACCME to provide continuing medical education for physicians.

Medscape, LLC designates this Journal-based CME activity for a maximum of 1 ***AMA PRA Category 1 Credit(s)™***. Physicians should claim only the credit commensurate with the extent of their participation in the activity.

All other clinicians completing this activity will be issued a certificate of participation. To participate in this journal CME activity: (1) review the learning objectives and author disclosures; (2) study the education content; (3) take the post-test with a 75% minimum passing score and complete the evaluation at www.medscape.org/journal/pcd; (4) view/print certificate.

**Release date: December 31, 2015; Expiration date: December 31, 2016**

### Learning Objectives

Upon completion of this activity, participants will be able to:

1. Assess some of the provisions of the Affordable Care Act and the health effects of insurance coverage
2. Distinguish the rate of not having health insurance among US adults in the current study
3. Evaluate characteristics of adults in states that have elected not to expand Medicaid
4. Analyze how Medicaid expansion has affected rates of health insurance coverage across states

**EDITOR**

Rosemarie Perrin, Editor, *Preventing Chronic Disease*. Disclosure: Rosemarie Perrin has disclosed no relevant financial relationships.

**CME AUTHOR**

Charles P. Vega, MD, Clinical Professor of Family Medicine, University of California, Irvine

Disclosure: Charles P. Vega, MD, has disclosed the following relevant financial relationships:

Served as an advisor or consultant for: Lundbeck, Inc.; McNeil Pharmaceuticals; Takeda Pharmaceuticals North America, Inc.

**AUTHORS**

Catherine A. Okoro, PhD, Population Health Surveillance Branch, Division of Population Health, National Center for Chronic Disease Prevention and Health Promotion, Centers for Disease Control and Prevention, Atlanta, Georgia

Disclosure: Catherine A. Okoro, PhD, has disclosed no relevant financial relationships.

 

Guixiang Zhao, MD, PhD, Population Health Surveillance Branch, Division of Population Health, National Center for Chronic Disease Prevention and Health Promotion, Centers for Disease Control and Prevention, Atlanta, Georgia

Disclosure: Guixiang Zhao, MD, PhD, has disclosed no relevant financial relationships.

 

Satvinder S. Dhingra, MPH, Northrop Grumman Corporation, Atlanta, Georgia

Disclosure: Satvinder S. Dhingra, MPH, has disclosed no relevant financial relationships.

 

Fang Xu, PhD, Population Health Surveillance Branch, Division of Population Health, National Center for Chronic Disease Prevention and Health Promotion, Centers for Disease Control and Prevention, Atlanta, Georgia

Disclosure: Fang Xu, PhD, has disclosed no relevant financial relationships.

## Introduction

The 2010 Affordable Care Act (ACA) (1,2) was intended to increase the number of Americans who could get health insurance (3). Under the ACA, every state was to expand Medicaid to residents with incomes at or below 138% of the federal poverty level (3,4). However, in June 2012, the US Supreme Court ruled that the ACA’s federal mandate to expand Medicaid was unconstitutional: states therefore had a choice in whether to expand eligibility to Medicaid (5). Health insurance through the ACA’s Health Insurance Marketplace became available on January 1, 2014, and many states expanded Medicaid effective on that date (6). From the start of the ACA open enrollment period in October 2013 through September 12, 2015, an estimated 15.3 million previously uninsured adults aged 18 to 64 years gained health insurance under this law (7).

Expanding access to health insurance lowers the financial barrier to health care in the United States and thus is intended to improve population health (8–12). Studies demonstrate that lower uninsured rates are associated with increased access to health care services and improvements in self-reported health, clinical depression, and mortality (8–12). Wallace and Sommers (8) used data from the 2005−2012 Behavioral Risk Factor Surveillance System (BRFSS) survey to examine the impact of the ACA on overall health and access to health care, particularly the dependent-coverage provision that allows young adults aged 19 to 25 years to be covered under their parents’ plans. Wallace and Sommers found that the dependent-coverage provision was associated with significant improvement in self-reported health in the intervention group of young adults aged 19 to 25 years compared with a control group of adults aged 26 to 34 years who were unaffected by the provision (8). The dependent coverage provision was associated with a 6.6 percentage point increase in health insurance among adults aged 19 to 25 years and a 0.8 percentage point decrease in the probability of self-reporting fair or poor health (8). Similar results in self-reported health were found among low-income adults who acquired Medicaid coverage (eg, an increase in the probability of screening negative for depression) (9,11). After Massachusetts enacted the 2006 Massachusetts Health Care Reform Law in April 2006, all-cause mortality and mortality from causes potentially treatable with timely care decreased by 2.9% and 4.5%, respectively, in that state compared with a control group of counties in nonreform states (ie, states pre-ACA enactment without their own health care reform laws) with population characteristics similar to the Massachusetts population (12). These reductions in Massachusetts were concentrated in populations in counties most likely to benefit from expanded access to health care, such as those in counties with high prereform uninsured rates and low incomes (12). Researchers studying 2000 through 2005 expansions of Medicaid in Arizona, Maine, and New York found similar increased access to health care, improved self-rated health, and reduced mortality (11).

Benefits associated with the ACA may vary among states because of differences in sociodemographic characteristics, available resources, and their populations’ socio-cultural-political environment. For example, as of September 2015, 31 states, including the District of Columbia, had begun Medicaid expansion; 1 state (Utah) was undecided; and 19 states chose not to expand Medicaid (13). In states that do not expand Medicaid, many low-income residents cannot afford health care coverage on the ACA health insurance exchanges and have incomes too high to qualify for Medicaid. This coverage gap means that low-income residents of these states continue to face a cost barrier to health insurance and, as a result, are at increased risk for poor health outcomes (8–12). Conversely, low-income residents of states that are expanding Medicaid have increased access to health care and, as a result, are expected to have improved health outcomes (8–12).

National and regional estimates of the prevalence of lack of health insurance among adults aged 18 to 64 years are available (6,14–16), but limited information exists regarding pre-ACA Medicaid expansion estimates at the state level to monitor and evaluate changes in health care access post-ACA Medicaid expansion (15,17,18). Thus, to add to that knowledge base, we used state data from 2013 BRFSS survey to estimate the state and national prevalence of lack of health insurance among adults aged 18 to 64 years. We describe the sociodemographic characteristics and Medicaid expansion status of states sorted into 3 groups: states with a low percentage of uninsured residents compared with the national average of 21.5% (95% confidence interval, 21.1%–21.8%) (high-insured states [<21.5%]), states with an average number of uninsured residents (average-insured states [≈ 21.5%]), and states with a high percentage of uninsured residents (low-insured states [>21.5%]).

## Methods 

BRFSS is an ongoing, dual-frame (landline telephone and cellular telephone), state-based survey conducted in all 50 states, the District of Columbia, and selected US territories. The purpose of BRFSS is to collect health information on the leading causes of disease and death among community-dwelling adults aged 18 years or older. Details about BRFSS survey design, sampling methods, data collection, and weights are available online (http://www.cdc.gov/brfss/) and are described elsewhere (19). In 2013, the American Association of Public Opinion Research (AAPOR) response rate for the BRFSS ranged from 29.0% in Alabama to 59.2% in North Dakota (median, 45.9%), and the AAPOR cooperation rate (the percentage of eligible people contacted who completed the interview) ranged from 47.5% in Alabama to 75.9% in both Colorado and Kentucky (median, 65.7%) (20).

Lack of health insurance was assessed by asking respondents whether they had any kind of health insurance, including health insurance, prepaid plans such as HMOs, government plans such as Medicare, or coverage through the Indian Health Service (IHS). We limited our analysis to adults aged 18 to 64 years because most adults aged 65 years or older are covered by Medicare.

We examined respondents’ sociodemographic characteristics by age (18−24, 25−44, and 45−64 y), sex, race/ethnicity (non-Hispanic white, non-Hispanic black, Hispanic, and other), educational attainment (≤ high school, some college, and ≥ college), marital status (married, previously married [divorced, widowed, and separated], never married, or member of an unmarried couple), employment status (employed, unemployed, or not in labor market — including homemaker, student, retired, and unable to work), annual household income ($0−$14,999, $15,000−$24,999, $25,000−$34,999, $35,000−$49,999, $50,000−$74,999, ≥$75,000, or unknown), presence of children and youths in the household younger than 18 years (yes, no, or unknown), and self-reported disability based on a yes response to either of 2 questions: “Are you limited in any way in any activities because of physical, mental, or emotional problems?” and “Do you now have any health problem that requires you to use special equipment, such as a cane, a wheelchair, a special bed, or a special telephone?”

For statistical analysis we first estimated the prevalence of lack of health insurance and adjusted it to the 2000 projected population for the United States and for each state. Second, states were sorted into 3 categories: 1) high-insured states with a prevalence of lack of health insurance below the national average (nonoverlapping 95% confidence intervals [CIs]); 2) average-insured states with an estimated prevalence of lack of health insurance equivalent to the national average (overlapping 95% CIs); and 3) low-insured states with an estimated prevalence of lack of health insurance above the national average (nonoverlapping 95% CIs). Third, we used bivariate analyses to compare the age-adjusted sociodemographic characteristics among these 3 groups. Finally, we examined the distribution of Medicaid expansion among the 3 groups. Both SAS (SAS Institute) and SUDAAN (RTI International) were used for all analyses to account for the complex sampling design, to estimate unadjusted and age-standardized prevalence and 95% CIs, and to test for statistical significance. All statistical inferences were based on a significance level of *P* < .05.

BRFSS was reviewed by the Human Research Protection Office of the Centers for Disease Control and Prevention (CDC) and determined to be exempt research.

## Results

In 2013, adults aged 18 to 64 years (N = 325,179) completed the BRFSS interview in all 50 states and the District of Columbia. After excluding from the analyses data on participants who responded “don’t know” or “not sure,” refused to answer, or had missing responses for current health insurance or sociodemographic variables (except for income and number of children in the household), data on 307,247 respondents remained for our analyses. These data were weighted to more than 183 million US adults aged 18 to 64 years (145 million adults with health insurance and 38 million without health insurance).

**Figure 1:**
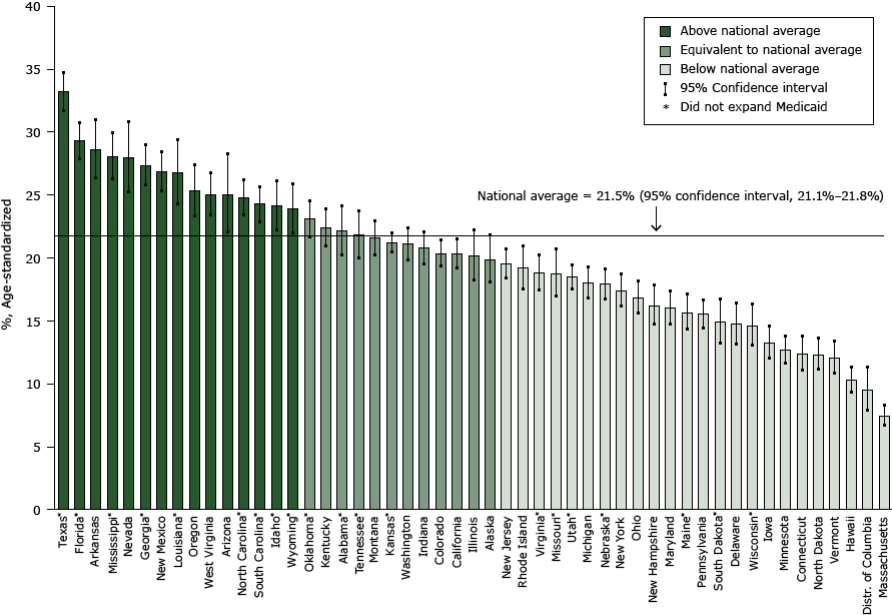
Estimated state prevalence of lack of health insurance in relation to the national average among adults aged 18 to 64 years, 2013 Behavioral Risk Factor Surveillance System (http://www.cdc.gov/brfss/). Asterisk indicates states that did not expand Medicaid. **Table:** 

Among US adults aged 18 to 64 years, the unadjusted prevalence of lack of health insurance was 20.8% (95% CI, 20.5%–21.1%) and the age-adjusted prevalence was 21.5% (95% CI, 21.1%–21.8%). The unadjusted and age-adjusted prevalence of lack of health insurance were lowest in Massachusetts (unadjusted prevalence, 7.1% [95% CI, 6.3%–8.1%]; adjusted prevalence, 7.5% [95% CI; 6.6%–8.5%]) and highest in Texas (unadjusted prevalence, 32.7% [95% CI, 31.0%–34.4%]; adjusted prevalence, 33.2% [95% CI, 31.5%–34.9%]) (Figure 1).

State	Age-Standardized Prevalence of Lack of Health Insurance, % (95% Confidence Interval)
**Above national average**	
Texas*	33.2 (31.5–34.9)
Florida*	29.3 (27.8–30.9)
Arkansas	28.6 (26.3–31.1)
Mississippi*	28.0 (26.1–30.0)
Nevada	28.0 (25.2–31.0)
Georgia*	27.3 (25.7–29.1)
New Mexico	26.9 (25.2–28.6)
Louisiana*	26.8 (24.2–29.5)
Oregon	25.3 (23.2–27.5)
West Virginia	25.0 (23.3–26.9)
Arizona	25.0 (21.9–28.4)
North Carolina*	24.8 (23.3–26.3)
South Carolina*	24.3 (22.8–25.8)
Idaho*	24.1 (22.1–26.3)
Wyoming*	23.9 (21.9–26.0)
**Equivalent to national average**	
Oklahoma*	23.1 (21.6–24.6)
Kentucky	22.4 (20.9–24.0)
Alabama*	22.1 (20.1–24.3)
Tennessee*	21.8 (19.9–23.9)
Montana	21.6 (20.1–23.1)
Kansas*	21.2 (20.4–22.1)
Washington	21.1 (19.8–22.5)
Indiana	20.8 (19.4–22.2)
Colorado	20.4 (19.2–21.5)
California	20.3 (19.1–21.6)
Illinois	20.2 (18.1–22.4)
Alaska	19.9 (18.0–21.9)
**Below national average**	
New Jersey	19.5 (18.3–20.9)
Rhode Island	19.2 (17.5–21.1)
Virginia*	18.8 (17.4–20.3)
Missouri*	18.7 (16.8–20.8)
Utah*	18.5 (17.4–19.6)
Michigan	18.0 (16.7–19.4)
Nebraska*	17.9 (16.6–19.2)
New York	17.4 (16.0–18.8)
Ohio	16.9 (15.5–18.3)
New Hampshire	16.2 (14.6–18.0)
Maryland	16.0 (14.7–17.5)
Maine*	15.7 (14.2–17.2)
Pennsylvania	15.5 (14.4–16.8)
South Dakota*	14.9 (13.1–16.8)
Delaware	14.7 (13.1–16.5)
Wisconsin*	14.6 (12.9–16.4)
Iowa	13.2 (11.9–14.7)
Minnesota	12.7 (11.5–13.9)
Connecticut	12.4 (11.0–13.9)
North Dakota	12.3 (11.0–13.7)
Vermont	12.1 (10.7–13.6)
Hawaii	10.3 (9.2–11.5)
District of Columbia	9.5 (7.8–11.5)
Massachusetts	7.5 (6.6–8.5)
**National average**	21.5 (21.1–21.8)

Compared with the national age-adjusted prevalence of lack of health insurance (21.5%), 24 states had lower rates of uninsured (ranging from 7.5% in Massachusetts to 19.5% in New Jersey); 12 states had equivalent rates of uninsured (ranging from 19.9% in Alaska to 23.1% in Oklahoma); and 15 states had higher rates of uninsured (ranging from 23.9% in Wyoming to 33.2% in Texas) (Figure 1).

Compared with adults in the high-insured or average-insured states, those in the low-insured states were more likely to be non-Hispanic black or Hispanic, to have no more than a high school education, to be previously married (divorced, widowed, separated), and to have an annual household income of $15,000 to $34,999 (Table). Compared with adults in the low-insured or average-insured states, adults in the high-insured states were more likely to be aged 45 to 64 years, to be non-Hispanic white, to have at least a college education, to have never married, to be employed, and to have annual household incomes of $50,000 or higher; they were also less likely to have children living at home. Additionally, adults in the high-insured states were less likely to be disabled than those in the average-insured states (17.5% vs 18.5%; *P* < .01).

**Table:** Sociodemographic Characteristics of Adults Aged 18 to 64 Years Categorized by Estimated State Prevalence of Lack of Health Insurance Coverage Compared With the US National Average, 2013 Behavioral Risk Factor Surveillance System (n = 307,247 [Unweighted])

Characteristic^a^	State Group, Weighted % (95% Confidence Interval)			*P* Value, Comparison of State Groups		
	A: High-Insured States^b^	B: Average-Insured States^c^	C: Low-Insured States^d^	A and B	C and B	A and C
**Age, y**						
18–24	15.5 (15.1–15.9)	15.8 (15.2–16.4)	15.6 (15.1–16.2)	.40	.67	.70
25–44	40.3 (39.8–40.7)	42.0 (41.3–42.7)	42.3 (41.6–43.0)	<.001	.52	<.001
45–64	44.2 (43.8–44.7)	42.2 (41.5–42.9)	42.0 (41.4–42.7)	<.001	.52	<.001
**Female**	50.6 (50.1–51.0)	50.1 (49.4–50.9)	50.5 (49.8–51.2)	.38	.46	.96
**Race/ethnicity**						
White, non-Hispanic	70.1 (69.6–70.6)	58.5 (57.7–59.2)	55.0 (54.3–55.7)	<.001	<.001	<.001
Black, non-Hispanic	11.7 (11.4–12.1)	8.7 (8.2–9.1)	16.5 (16.0–17.0)	<.001	<.001	<.001
Hispanic	10.3 (10.0–10.6)	21.4 (20.7–22.1)	22.7 (22.1–23.4)	<.001	<.01	<.001
Other, non-Hispanic^e^	7.9 (7.6–8.2)	11.4 (10.8–12.1)	5.7 (5.4–6.1)	<.001	<.001	<.001
**Education**						
≤High school	39.0 (38.5–39.5)	41.4 (40.6–42.1)	44.4 (43.7–45.1)	<.001	<.001	<.001
Some college	31.1 (30.6–31.5)	32.0 (31.3–32.7)	32.1 (31.4–32.7)	<.05	.83	<.05
≥College	30.0 (29.6–30.3)	26.7 (26.1–27.2)	23.5 (23.0–24.0)	<.001	<.001	<.001
**Marital status**						
Married	50.6 (50.2–51.0)	51.2 (50.5–51.9)	50.5 (49.9–51.1)	.15	.15	.81
Previously married^f^	13.5 (13.2–13.8)	15.0 (14.6–15.5)	16.8 (16.3–17.3)	<.001	<.001	<.001
Never married/member of an unmarried couple	35.9 (35.5–36.3)	33.8 (33.2–34.4)	32.7 (32.1–33.3)	<.001	<.05	<.001
**Employment status**						
Employed	69.2 (68.7–69.6)	64.8 (64.1–65.5)	64.6 (64.0–65.3)	<.001	.63	<.001
Unemployed	8.4 (8.1–8.7)	9.2 (8.7–9.6)	9.0 (8.6–9.4)	<.01	.64	<.05
Not in labor market^g^	22.5 (22.1–22.9)	26.0 (25.4–26.6)	26.4 (25.8–27.0)	<.001	.40	<.001
**Annual household income, $**						
0–14,999	9.3 (9.0–9.6)	13.3 (12.8–13.9)	12.1 (11.7–12.6)	<.001	<.01	<.001
15,000–24,999	13.7 (13.3–14.0)	14.4 (13.9–15.0)	17.5 (16.9–18.0)	<.05	<.001	<.001
25,000–34,999	8.4 (8.2–8.7)	8.9 (8.5–9.3)	10.0 (9.6–10.4)	.08	<.001	<.001
35,000–49,999	11.8 (11.5–12.1)	11.8 (11.4–12.3)	12.2 (11.8–12.7)	.97	.28	.17
50,000–74,999	14.1 (13.7–14.4)	13.3 (12.8–13.8)	12.2 (11.8–12.7)	<.05	<.01	<.001
≥75,000	31.3 (30.8–31.7)	28.1 (27.5–28.8)	23.6 (23.0–24.2)	<.001	<.001	<.001
Unknown	11.5 (11.2–11.9)	10.1 (9.6–10.5)	12.4 (11.9–12.8)	<.001	<.001	<.01
**≥1 Children in household**	46.3 (45.9–46.8)	47.9 (47.2–48.7)	48.0 (47.3–48.6)	<.001	.92	<.001
**Self-reported disability**	17.5 (17.2–17.9)	18.5 (18.0–19.1)	18.1 (17.6–18.5)	<.01	.18	.07

a Sociodemographic characteristics (except age) were age-adjusted to the 2000 US standard population.

b States with an estimated prevalence of lack of health insurance less than the lower bound of the 95% CI of the national average are Connecticut, Delaware, Hawaii, Iowa, Maine, Maryland, Massachusetts, Michigan, Minnesota, Missouri, Nebraska, New Hampshire, New Jersey, New York, North Dakota, Ohio, Pennsylvania, Rhode Island, South Dakota, Utah, Vermont, Virginia, Wisconsin, and the District of Columbia (24 states; n = 148,352).

c States with an estimated prevalence of lack of health insurance coverage within the 95% CI of the national average are Alabama, Alaska, California, Colorado, Illinois, Indiana, Kansas, Kentucky, Montana, Oklahoma, Tennessee, and Washington (12 states; n = 78,289).

d States with an estimated prevalence of lack of health insurance coverage greater than the upper bound of the 95% CI of the national average are Arizona, Arkansas, Florida, Georgia, Idaho, Louisiana, Mississippi, Nevada, New Mexico, North Carolina, Oregon, South Carolina, Texas, West Virginia, and Wyoming (15 states; n = 80,606).

e “Other” includes respondents who reported their ethnicity as non-Hispanic and their race as American Indian or Alaska Native, Asian, Native Hawaiian or Pacific Islander, some other race, or more than 1 race (ie, multiracial).

f “Previously married” includes respondents who reported their marital status as divorced, widowed, or separated.

g “Not in labor market” includes respondents who reported their employment status as a student, a homemaker, retired, or unable to work.

**Figure 2:**
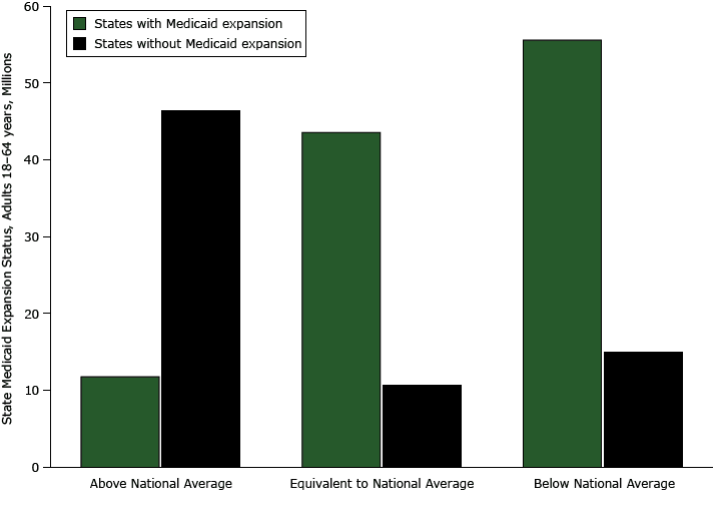
Population of adults aged 18 to 64 years by Medicaid expansion status among 3 state groups (above national average, equivalent to national average, below national average) categorized by estimated state prevalence of lack of health insurance in relation to the national average, 2013 Behavioral Risk Factor Surveillance System (http://www.cdc.gov/brfss/). Fifteen states were above the national average (6 with Medicaid expansion and 9 without); 12 states were equivalent to the national average (8 with Medicaid expansion and 4 without), and 24 states were below the national average (17 with Medicaid expansion and 7 without). **Table:** 

Seventeen of the 24 (70.8%) high-insured states expanded Medicaid eligibility compared with 8 of the 12 (66.6%) average-insured states and 6 of the 15 (40.0%) the low-insured states. Approximately 46.5 million adults aged 18 to 64 years reside in low-insured states that have opted out of Medicaid expansion compared with 10.7 million in average-insured states and 15.0 million in high-insured states and may be affected by states’ decisions not to expand Medicaid (Figure 2).

Medicaid Expansion Status, US Adults Aged 18–64 Years	Above National Average, Millions	Equivalent to National Average, Millions	Below National Average, Millions
Live in state with expanded Medicaid	11.8	43.6	55.6
Live in state without expanded Medicaid	46.5	10.7	15.0
Total	58.3	54.3	70.6

## Discussion

We used data from the 2013 BRFSS, the largest and longest-running public health, state-based, random-digit-dialed surveillance system in the United States, to estimate lack of health insurance at the state level and nationwide pre-ACA Medicaid expansion. These estimates provide a baseline with which to monitor changes in health care access post-ACA and expansion of Medicaid eligibility within states. We reported several key findings. First, after sorting states into 3 groups (those with lower levels of health insurance than the national average, those with equivalent levels, and those with higher levels), we found significant differences in sociodemographic characteristics among these groups. Adults in the low-insured states were more likely to be non-Hispanic black or Hispanic, to have low educational attainment, to have incomes between $15,000 and $34,999, and to be previously married than adults in the average-insured and high-insured states. Second, a higher proportion of states in the high-insured group expanded Medicaid eligibility than the proportion of states in the low-insured and average-insured groups. Finally, approximately 46.5 million adults reside in the low-insured states that are not expanding Medicaid.

Our study’s estimate of a national uninsured rate of 20.8% in 2013 is consistent with findings of other national surveys (15,17,21). By using the Gallup-Healthways Well-Being Index, Sommers et al (21) estimated the national unadjusted uninsured rate to be 21.0% in September 2013. The American Community Survey (ACS) estimate was 20.3% (17), and the 2013 National Health Interview Survey (NHIS) estimate was 20.4% (15). Notably, Sommers et al (21) reported that, by the second quarter of 2014, the uninsured rate declined 6.0 percentage points (*P* < .01) among low-income adults who reside in states that expanded Medicaid eligibility whereas uninsured rates among low-income adults who reside in states that did not expand eligibility declined only 3.1 percentage points (*P* = .13). Approximately 1 year later, uninsured rates declined among low-income adults residing in both Medicaid expansion states (13.0 percentage points) and nonexpansion states (7.0 percentage points) (22).

In 2014, the ACS (17) published estimates of the number of uninsured Americans nationwide, and the NHIS (15) published estimates for 43 states. These estimates were similar to the BRFSS’s state estimates for 2014 with some differences. Although all 3 surveys found large variations in the unadjusted uninsured rate by state, state rankings varied from one survey to the other. NHIS state estimates ranged from 4.7% for the District of Columbia to 29.3% for Nevada; the ACS range was 5.2% for Massachusetts to 29.9% for Texas, and the BRFSS range was 7.1% for Massachusetts to 32.7% for Texas. Estimates derived from these 3 surveys may vary for several reasons, such as differences in survey methodology, sample size, and precision; mode of interview; and various question effects (eg, order, number, wording, probes). Additionally, in contrast to these surveys, the BRFSS includes IHS health insurance. The impact of the inclusion of IHS coverage on our estimates needs further elucidation, particularly for states with large populations of American Indians or Alaska Natives. Our results regarding the sociodemographic characteristics of adults residing in states with low and high uninsured rates were consistent with the existing body of scientific literature (15,21,23). We found a disproportionately high number of adults aged 18 to 64 years residing in states that did not expand Medicaid. Low-income residents in these states may not be able to afford health insurance through the ACA’s Health Insurance Marketplace and have incomes too high to qualify for Medicaid. This coverage gap means that low-income adults of these states continue to face a financial barrier to health insurance and, as a result, remain at increased risk for poor health outcomes (8–12). The impact of states’ Medicaid expansion decisions may also vary based on the racial/ethnic distribution of adults in each state because Hispanics and non-Hispanic blacks are more likely to lack health insurance than non-Hispanic whites (27% and 16%, respectively, vs 11%) (23). Notably, 7 of the 9 states that chose not to expand Medicaid in the low-insured group are Southern states. Given that non-Hispanic blacks are a large proportion of the southern population, and are more likely to be uninsured, they are estimated to account for a disproportionate share of those in the coverage gap (24% vs 11% of non-Hispanic white uninsured adults and 7% of Hispanic uninsured adults), which results in increased racial disparities in health (23). Conversely, several states with large Hispanic populations (eg, Arizona, California, and New York) expanded Medicaid and thus increased access to health insurance and services for their eligible uninsured Hispanic adults (23).

The federal government finances 100% of Medicaid expansion of ACA for the first 3 years. Federal financing then tapers off to 95% in 2017 and 90% by 2021 (24). In addition to receiving the initial federal financing, states may save approximately $18 billion in uncompensated health care costs through 2022 (24,25). Despite these potential benefits, 19 states chose not to expand Medicaid (13). Some states, such as Tennessee and Utah, are exploring alternative Medicaid expansion plans, such as through private health plans (as in Arkansas and Michigan), cost-sharing, or incentives for healthy behaviors (13,26). Research has demonstrated, however, that cost-sharing can deter people from accessing needed health care, particularly low-income people (27). States that expanded Medicaid for ACA are seeing benefits such as reductions in state Medicaid program costs and lower numbers of uninsured residents. Post-expansion savings for the state budget in Michigan, for example, were projected to cover increased health care costs through 2027 (28).

This study has limitations. BRFSS data are self-reported, and self-report is subject to bias. However, the question used to estimate number of uninsured produced estimates comparable with those from the NHIS and the National Health and Nutrition Examination Survey (29). Sociodemographic characteristics and state of residence are only a few of many factors that may be associated with lack of health insurance. This study did not examine other possible factors such as attitudes, awareness, and knowledge of the ACA; health status; and state-specific determinants (eg, available resources, sociocultural and political climate). Additionally, this analysis did not examine the impact of pre-2013 state Medicaid expansions on study results (30). Finally, the BRFSS is limited to community-dwelling adults, excluding certain populations that are likely to have access to health care, such as institutionalized adults and those in the US Armed Forces.

The number of uninsured people younger than 65 years is expected to drop from 55 million in 2013 to 29 million in 2016 (16). The remaining uninsured population will comprise unqualified immigrants (who are not eligible for health insurance under ACA); those who are ineligible for Medicaid because they reside in a state that did not expand Medicaid; those who are eligible for Medicaid but choose not to enroll; and those who choose to remain uninsured, although they have access to insurance through an employer, an exchange, or directly from an insurer (16,23,24).

To the best of our knowledge, this is the first study to use BRFSS data to estimate the number of people without health insurance pre-Medicaid expansion by state and to examine the sociodemographic characteristics and states’ Medicaid expansion status in relation to these estimates. When 2014 BRFSS data become available, it will be possible to assess changes in health care access following ACA’s coverage expansion at the state level. Our results show large variations among states in the estimated prevalence of lack of health insurance. Of note, a large number of uninsured adults aged 18 to 64 years reside in states that opted out of Medicaid expansion. Continued surveillance at the state level is needed to monitor the effect of the ACA and Medicaid expansion on health care access, use of health services, health outcomes, and their economic impact in multiple health care domains.

## Figures and Tables

**Table:** 

**1. The activity supported the learning objectives.**				
**Strongly Disagree**				**Strongly Agree**
1	2	3	4	5
**2. The material was organized clearly for learning to occur.**				
**Strongly Disagree**				**Strongly Agree**
1	2	3	4	5
**3. The content learned from this activity will impact my practice.**				
**Strongly Disagree**				**Strongly Agree**
1	2	3	4	5
**4. The activity was presented objectively and free of commercial bias.**				
**Strongly Disagree**				**Strongly Agree**
1	2	3	4	5

## Post-Test Information

To obtain credit, you should first read the journal article. After reading the article, you should be able to answer the following, related, multiple-choice questions. To complete the questions (with a minimum 75% passing score) and earn continuing medical education (CME) credit, please go to http://www.medscape.org/journal/pcd. Credit cannot be obtained for tests completed on paper, although you may use the worksheet below to keep a record of your answers. You must be a registered user on Medscape.org. If you are not registered on Medscape.org, please click on the "Register" link on the right hand side of the website to register. Only one answer is correct for each question. Once you successfully answer all post-test questions you will be able to view and/or print your certificate. For questions regarding the content of this activity, contact the accredited provider, CME@medscape.net. For technical assistance, contact CME@webmd.net. American Medical Association's Physician's Recognition Award (AMA PRA) credits are accepted in the US as evidence of participation in CME activities. For further information on this award, please refer to http://www.ama-assn.org/ama/pub/about-ama/awards/ama-physicians-recognition-award.page. The AMA has determined that physicians not licensed in the US who participate in this CME activity are eligible for ***AMA PRA Category 1 Credits*** ™. Through agreements that the AMA has made with agencies in some countries, AMA PRA credit may be acceptable as evidence of participation in CME activities. If you are not licensed in the US, please complete the questions online, print the AMA PRA CME credit certificate and present it to your national medical association for review.

## Post-Test Questions

### Article Title: Lack of Health Insurance Coverage Among Adults Aged 18 to 64 Years: Findings From the 2013 Behavioral Risk Factor Surveillance System

**CME Questions**

1. Which of the following statements regarding expanding health insurance coverage and the Affordable Care Act (ACA) is ***most*** accurate?
  - The ACA has enrolled nearly 1 million previously uninsured adults in health insurance between 2013 and 2015
  - Health insurance coverage is associated with improved access to healthcare services, improved self-reported health, and reduced mortality rates
  - Medicaid expansion has had little effect on the rate of uninsured among poor adults
  - States will have to pay for 75% of Medicaid expansion by 2021
2. What was the age-adjusted rate of lack of health insurance among US adults 18 to 64 years old in the current study?
  - 2.6%
  - 5.9%
  - 10.0%
  - 21.5%
3. Which of the following variables was a characteristic associated with living in a non-Medicaid expansion state in the current study?
  - Non-Hispanic black or Hispanic race/ethnicity
  - Higher educational attainment
  - Never-married status
  - Household income above $50,000
4. Which of the following statements comparing non-Medicaid expansion states vs Medicaid expansion states in the current study is ***most*** accurate?
  - Most adults lived in non-Medicaid expansion states
  - Most adults lived in states with high rates of health insurance coverage
  - Medicaid expansion was paradoxically associated with categorization as a low-insured state
  - Medicaid expansion failed to affect overall health insurance coverage status in comparing different states

**Evaluation**
